# The Antimicrobial Peptide Nal-P-113 Exerts a Reparative Effect by Promoting Cell Proliferation, Migration, and Cell Cycle Progression

**DOI:** 10.1155/2018/7349351

**Published:** 2018-09-12

**Authors:** Nana Liu, Shuo Guan, Hongyan Wang, Chen Li, Jyawei Cheng, Huiyuan Yu, Li Lin, Yaping Pan

**Affiliations:** ^1^Department of Periodontics, School of Stomatology, China Medical University, Shenyang 110002, China; ^2^Department of Periodontics, Suzhou Stomatological Hospital, Stomatological Hospital Affiliated to Soochow University, Suzhou 215008, China; ^3^Department of Stomatology, The First Affiliated Hospital of Dalian Medical University, Dalian 116000, China; ^4^Institute of Biotechnology and Department of Medical Science, National Tsing Hua University, Hsinchu 300, Taiwan

## Abstract

**Objective:**

The primary purpose of this study was to evaluate the reparative efficacy of a novel antimicrobial peptide, Nal-P-113, in shortening the healing time of oral mucosal ulcers by promoting cell proliferation and migration and accelerating the cell cycle.

**Methods:**

Cell counting kit-8 (CCK-8) and wound-healing assays were used to evaluate the proliferation and migration of human immortalized oral epithelial cells (HIOECs). The cell cycle distribution of HIOECs was analyzed by flow cytometry. Additionally, the RNA levels of EGF, FGF-2, and TGF-*β*1 of HIOECs were assessed by real-time PCR. Rats were divided into three groups randomly: (a) blank control group; (b) 20 *μ*g/mL Nal-P-113; and (c) 10 ng/mL rhEGF. An oral mucosal ulcer was induced in every rat by the application of 30% acetic acid. An immunohistochemical assay was used to assess the expression of EGF, FGF-2, and TGF-*β*1 in the rat oral mucosa.

**Results:**

In the CCK-8 assay, the optical density values in the Nal-P-113 and rhEGF groups were found to be significantly higher than that in the blank control group. In addition, the scratch areas in the Nal-P-113 and rhEGF groups were found to be significantly smaller (*P*<0.05). Cell cycle analysis showed that Nal-P-113 accelerated the entry of HIOECs into the S phase and expedited their cell cycles. The RT-PCR results suggested that Nal-P-113 upregulated the RNA levels of EGF and FGF-2 but downregulated that of TGF-*β*1 at 24 h and 48 h. Lastly, the immunohistochemical assay verified that Nal-P-113 changed the expression of the above cytokines in rat mucosal ulcers.

**Conclusion:**

Nal-P-113 promoted the repair of oral mucosal ulcers by increasing the EGF and FGF-2 expression and decreasing that of TGF-*β*1 in HIOECs, accelerating their proliferation and cell cycle progression. The application of Nal-P-113 might serve as an effective therapeutic approach for recurrent aphthous stomatitis.

## 1. Introduction

Recurrent aphthous stomatitis (RAS) is a common, chronic, ulcerative disease in the oral mucosa, whose incidence is approximately 5% to 20% [1]; its 3-month recurrence rate is close to 50% [2]. Individuals with RAS experience significant pain, discomfort, altered speaking ability, and impaired swallowing. However, to date, the etiology of RAS has not been clearly defined. Some studies showed that RAS is triggered by many factors, such as trauma, drugs, food allergies, nutrient deficiencies, mental stress, tobacco, and genetic and immune factors. Given its indeterminate pathogenesis, numerous treatments are available in clinical practice, including locally applied gels, creams, and ointments, and systemically used medicated mouthwashes [3]. Therapeutic medicines that effectively shorten the duration of RAS and accelerate the repair of damage to oral epithelial cells could significantly ease the physical and mental burden of affected patients. Therefore, the ideal remedial drugs for RAS should be easy to apply topically, have few and mild side effects, act as anti-infection agents, decrease inflammation, and facilitate tissue healing.

Recently, antimicrobial peptides (AMPs), which are natural antibacterial substances found in living organisms, have attracted significant research interest because antibiotic resistance has become increasingly prominent. AMPs are small-molecule proteins or peptides that protect against bacteria, fungi, and viruses [4]. Many studies have indicated that AMPs exist in almost all biological creatures and play a vital role in immune regulation as a part of the innate immune system or cause cells apoptosis [5, 6]. Unfortunately, the bioactivity of natural AMPs is undesirable, and the structure necessary for their biological performance is not stabilized. Therefore, the use of AMPs is less favorable in the medical field than in research [7]. A novel antimicrobial peptide, named Nal-P-113 (Ac-AKR-Nal-Nal-GYKRK F-Nal-NH_2_), was synthesized from P-113 (AKRHHGYKRKFH-NH2) by replacing histidines 4, 5, and 12 with *β*-naphthylalanines. Our previous study has confirmed that *β*-naphthylalanine residues could position themselves deeper into the bacterial and fungal cell membranes, making the antimicrobial peptide more efficient in disrupting the membranes. Furthermore, *β*-naphthylalaine also increases the stability of the antimicrobial peptide in high-salt conditions which provides application possibility for cytological and clinical studies [8, 9]. Meantime, we have found that Nal-P-113 at lower concentration did not have cytotoxicity to human periodontal ligament stem cells and gingival epithelial cells (epi4) by flow cytometry and confocal microscopy [9]. Some volunteers used Nal-P-113 gels as a periodontal antimicrobial agent in the clinical test, and they did not feel any discomfort in the process of application [10]. Nal-P-113 is, therefore, a synthetic antimicrobial peptide with high yield and excellent sterilization and antiviral effects, having great promise for broad application in the development and manufacture of improved antimicrobial agents.

Epidermal growth factor (EGF) is a 53-amino acid polypeptide that significantly contributes to the maintenance of the integrity of the gastrointestinal mucosa. EGF is suggested to exert cytoprotective effects against injuries to the oral mucosa membrane by recognizing the EGF receptor to activate the pathways that promote cell proliferation, survival, migration, and differentiation in most epithelial tissues, fibroblasts, and endothelial cells [11, 12]. Balicki R et al. thought that the signal transduction between EGF and the corresponding receptor consequently influences gene regulation by phosphorylating the receptor to amplify intrinsic tyrosine kinase activity [13]. Studies have used EGF as a positive control and biomarker to better understand the mechanisms of oral lichen planus and oral squamous cell carcinoma [14]. Fibroblast growth factor-2 (FGF-2), which is a member of the FGF family, is closely associated with the regulation of cell proliferation, migration, differentiation, apoptosis and the processes of neovascularization, normal wound healing, and angioproliferative diseases* in vitro* [15, 16]. Studies investigating hindlimb ischemia revealed that UCX® could upregulate the concurrent expression of a few proangiogenic factors, including FGF-2, in endothelial cells, which could, in turn, increase their angiogenic capacity [17]. Transforming growth factor-*β*1 (TGF-*β*1) is a member of the transforming growth factor beta superfamily of cytokines and can effectively regulate and control cell growth, proliferation, differentiation and apoptosis via signal transduction to alter the activation of TGF-*β* [18, 19]. Furthermore, TGF-*β*1 is a secreted protein that plays an important role in other critical processes, including embryonic development, wound healing, and immune regulation [20]. Previous studies have shown that TGF-*β*1 performs an important function in facilitating tubular epithelial-myofibroblast transdifferentiation by altering the phenotype and inhibiting the growth of renal epithelial cells [21].

In this study, we postulated that Nal-P-113 could shorten the healing time of oral mucosal ulcers by changing the expression of EGF, FGF-2, and TGF-*β*1 in human immortalized oral epithelial cells (HIOECs), which would accelerate the cell cycle and promote cell proliferation and migration. Nal-P-113 represents an auxiliary new approach for the therapy of RAS.

## 2. Materials and Methods

### 2.1. Preparation of the Antimicrobial Peptide Nal-P-113

Nal-P-113, Ac-AKR-Nal-Nal-GYKRKF-Nal-NH2, was synthesized from P-113 (AKRHHGYKRKFH-NH2) by replacing His 4, 5, and 12 with *β*-naphthylalanines based on the 9-fluorenylmethoxycarbonyl (Fmoc) solid-phase synthesis protocol [8, 22]. The identity of the peptides was confirmed by electrospray mass spectrometry, and the purity was assessed by high-performance liquid chromatography (HPLC). The peptide concentration was determined with a UV spectrophotometer (280 nm) [23].

### 2.2. Cell Culture

HIOECs were kindly supplied by the Key Laboratory of Stomatology, affiliated with the Ninth People's Hospital, Jiao Tong University School of Medicine in Shanghai. The HIOECs were cultured in defined keratinocyte serum-free medium (DK-SFM) (Gibco, CA, USA) in a humidified atmosphere of 5% CO_2_ at 37°C.

### 2.3. CCK-8 Assay

HIOECs in the logarithmic growth phase were seeded into a 96-well plate at a density of 5×10^3^ cells per 100 *μ*L and cultured in a 37°C and 5% CO_2_ atmosphere* in vitro*. After 24 h, the cell medium was entirely replaced with the blank control, Nal-P-113 or rhEGF (Pavay, Guangxi, China) to make the final concentrations of Nal-P-113 and rhEGF 20 *μ*g/mL and 10 ng/mL, respectively. CCK-8 reagent (Dinguo, Beijing, China) (100 *μ*L; CCK-8: culture medium=1:10) was added to each well at 6 h, 12 h, 24 h, 36 h, and 48 h, and the mixtures were incubated for 1-4 h at 37°C under light-free conditions. The absorbance value was measured at a wavelength of 450 nm using a microplate reader (Tecan, Salzburg, AT).

### 2.4. Wound-Healing Assay

A marker pen was used to create several uniform horizontal lines on the back of a 6-well plate at intervals of 0.5 cm, and at least 5 lines passed through each well. HIOECs in the logarithmic growth phase were seeded into each well at a density of 2×10^5^ cells. Then, a sterile P200 pipette tip was used to perpendicularly scratch the previous lines following previously described methods [24, 25]. These cells were washed with PBS 3 times and cultured with the blank control, Nal-P-113 or rhEGF at 37°C and 5% CO_2_. The final concentrations of Nal-P-113 and rhEGF were 20 *μ*g/mL and 10 ng/mL, respectively. Photographs were taken at 0 h and 48 h with NIS-Elements D 2.30 software (Nikon, Japan). The healing areas of specified scratches were assessed using Image-Pro Plus 5.1 image analysis software (Media Cybernetics, Silver Spring, MD) and the formula healing rate=(initial scratch area–present scratch area)/initial scratch area×100% [11].

### 2.5. Flow Cytometric Analysis

HIOECs in the logarithmic growth phase were seeded into a 6-well plate at a density of 2×10^5^ cells and cultured in a 37°C and 5% CO_2_ atmosphere. After 24 h, 20 *μ*L 2 mg/mL Nal-P-113 or 1 *μ*g/mL rhEGF was added to 1980 *μ*L cells medium, and the final concentrations of Nal-P-113 and rhEGF were 20 *μ*g/mL and 10 ng/mL, respectively, with treatments lasting for 48 h. Then, all HIOECs were treated with trypsin, washed with precooled PBS, and fixed in 70% ethanol at 4°C overnight. Ethanol-fixed cells were washed with PBS and treated with propidium iodide (50 *μ*g/mL) and RNase (100 *μ*g/mL) in PBS in the dark for 30 min. The cell cycle distribution of 1×10^4^ cells per sample was determined with excitation at 488 nm in a flow cytometer (FACS, Becton-Dickinson, Islandia, NY, USA). The data were analyzed with Cell-Quest software (BD Bioscience, Rockville, MD, USA) and ModFit LT V 3.1 (Verity software, Topsham, ME, USA).

### 2.6. Real-Time PCR Assay

HIOECs in the logarithmic growth phase were seeded into 6-well plates at a density of 2×10^5^ cells/cm^2^ and cultured in a medium containing the blank control, Nal-P-113 or rhEGF in a 37°C and 5% CO_2_ atmosphere, and the final concentrations of Nal-P-113 and rhEGF were 20 *μ*g/mL and 10 ng/mL, respectively. After 24 h and 48 h, all samples were collected to obtain the total RNA using a total RNA extraction kit (TaKaRa, Otus, Japan) according to the instructions. Then, the cDNA strand was synthesized according to the provided protocol using a real-time PCR instrument (Applied Biosystems, Foster City, CA, USA). The system procedures were as follows: 2 min at 95°C and then 40 cycles of 15 s at 95°C and 1 min at 60°C. The specific primers used in this study are listed in Table 1. The gene expression levels were evaluated using MxPro-Mx3000P software (Stratagene, La Jolla, CA, USA). The data were analyzed according to the relative gene expression by the 2^-ΔΔCt^ method. Genes with a fold change >1.5 were considered to be significantly differentially expressed [26].

### 2.7. Ethical Statement

The use of Sprague-Dawley rats in this study complied with Animal Research Reporting* In Vivo* Experiments (ARRIVE) guidelines. The experimental protocol was approved by the ethics committee of China Medical University.

### 2.8. Animals

Five-week-old, healthy, male Sprague-Dawley rats weighing approximately 200–250 g were used. All rats were fed on a 12 h light-dark cycle at 21±2°C room temperature and 60±5% humidity. Rats were randomly assigned to one of the following groups (8 rats per group): (a) blank control group (saline); (b) 20 *μ*g/mL of Nal-P-113; and (c) 10 ng/mL rhEGF. So far, no definite conclusions showed that physiological saline had an effect on promoting the ulcer healing and inhibiting the bacterial growth. In view of the above consideration, in this study, physiological saline was taken as a blank control.

The rat oral mucosal ulcer models were built in strict accordance with the modified methods previously described by Konturek et al. [27]. At day 0, all rats were anesthetized with 10% chloral hydrate, and 200 *μ*L of 30% acetic acid (Dinguo, Beijing, China) was applied to the oral mucosal surface of the rats for 30 s using a glass tube (inner diameter of 6 mm and depth of 30 mm) to induce ulcerative injury [28]. After 3 days, the ulcer wounds of each group were douched with 1 mL of saline, Nal-P-113 or rhEGF, on the 3rd, 5th, 8th and 10th days, respectively, with the rats receiving inhalational anesthesia with sevoflurane each time (Hengrui Pharmaceutical Co. Ltd., Shanghai, China).

### 2.9. Immunohistochemical Assay

On the 3^rd^, 5^th^, 8^th^, and 10^th^ days after rat ulceration, after sacrifice by spinal dislocation, buccal mucosal specimens were collected and fixed in 4% paraformaldehyde. These slices were deparaffinized and rehydrated, and the endogenous peroxidase activity was blocked by 3% H_2_O_2_ in deionized water at room temperature for 10 min. The samples were then boiled in citrate buffer (0.01 mol/L) for 20 min and blocked with sheep serum at 37°C for 10 min. Next, the slices were incubated with rabbit anti-rat EGF, FGF-2, and TGF-*β*1 polyclonal antibodies (Boshide, Wuhan, China) at 4°C overnight, then incubated with biotinylated sheep anti-rabbit IgG monoclonal antibodies at 37°C for 20 min, and finally incubated with the streptavidin-biotin complex working solution (SABC kit, Boshide, Wuhan, China) at 37°C for 20 min; the samples were washed fully with 0.1 M KPBS between each incubation step. After using a 3, 3'-diaminobenzidine chromogenic kit (Boshide, Wuhan, China), the oral mucosal sections were lightly counterstained with hematoxylin. Ten random fields of view from each sample were observed under an optical microscope (×100), and the average optical density values of EGF, FGF-2, and TGF-*β*1 were subsequently calculated by the Axioplan 2 microscopic image analysis system (UIC, USA).

### 2.10. Statistical Analysis

All the above experiments were performed in triplicate and repeated at least three times. The experimental data are expressed as the means ± standard deviations (SDs), and the variance was analyzed using SPSS 17.00 software. ANOVA with post hoc test was performed for analysis among the groups when the variances between two groups were different at a significance level of *α* = 0.05 and a 95% confidence level.

## 3. Results

### 3.1. HIOECs Proliferation, Detected by the CCK-8 Assay

**Figure 1:**
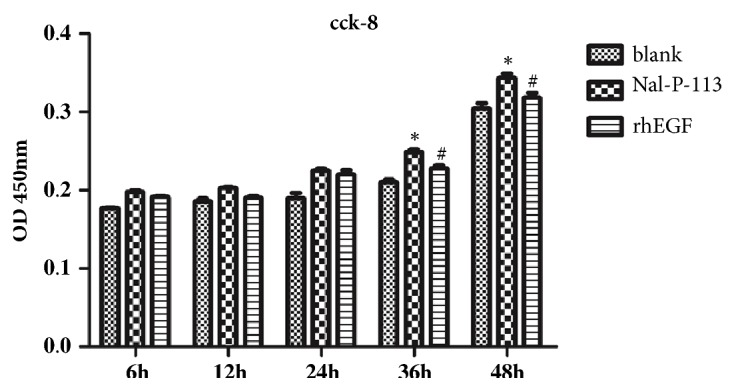
The proliferation of HIOECs was determined by the CCK-8 assay. HIOECs were treated with blank control, 20 *μ*g/mL Nal-P-113 or 10 ng/mL rhEGF at 6 h, 12 h, 24 h, 36 h, and 48 h. The data are presented as the mean±SD of three independent experiments. *∗*: significant differences (*P* < 0.05) between Nal-P-113 group and blank control group at the same time point. #: Significant differences (*P* < 0.05) between rhEGF group and blank control group at the same time point.

As shown in Figure 1, the proliferation rate of HIOECs treated by Nal-P-113 or rhEGF was not significantly different from that of the blank control group before 36 h (*P* > 0.05). However, 20 *μ*g/mL Nal-P-113 and 10 ng/mL EGF increased the proliferation rate of HIOECs at 36 h and 48 h (*P* < 0.05). According to these results, we thought that Nal-P-113 and rhEGF could promote the proliferation of HIOECs after 36 h.

### 3.2. Wound-Healing Assay

**Figure 2:**
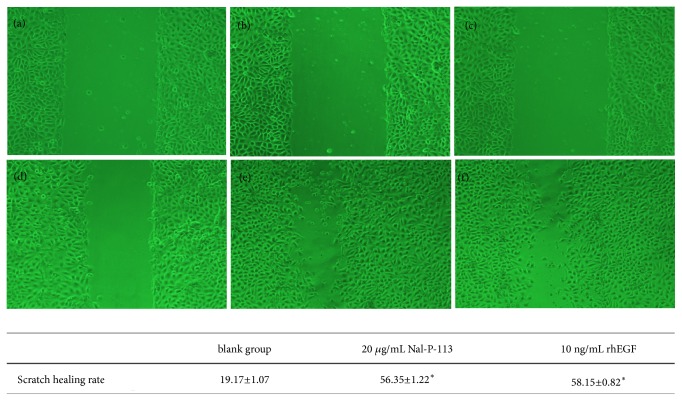
The wound healing rate was used to determine the migration changes of HIOECs at 48 h. *∗*: significant differences (*P* < 0.05) between Nal-P-113 or rhEGF group and blank control group at the same time point. (a, d) HIOECs in the blank control group were cultured for 48 h and photographed at 0 h and 48 h (×100). (b, e) HIOECs were treated with 20 *μ*g/mL Nal-P-113 for 48 h and photographed at 0 h and 48 h (×100). (c, f) HIOECs were treated with 10 ng/mL rhEGF for 48 h and photographed at 0 h and 48 h (×100).

As shown in Figure 2, the wound healing rate in the Nal-P-113 group at 48 h was 56.35±1.22, which was significantly different (*P* < 0.05) from that of the blank control group (19.17±1.07). At the same time, the value in the rhEGF group was 58.15±0.82, and the difference was statistically significant between the rhEGF group and the blank control group (*P*<0.05). The results suggested that Nal-P-113 and rhEGF could accelerate the migration of HIOECs at 48 h.

### 3.3. Nal-P-113 Causes HIOECs to Enter the S Phase Rapidly, Accelerating the Cell Cycle

**Figure 3:**
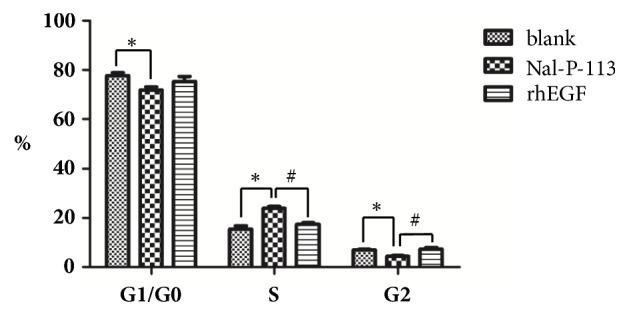
The effects of 20 *μ*g/mL Nal-P-113 and 10 ng/mL rhEGF on the cell cycle distribution of HIOECs were evaluated by flow cytometry. *∗*: there were significant differences (*P* < 0.05) between Nal-P-113 group and blank control group at three phases. #: significant differences (*P* < 0.05) between Nal-P-113 group and rhEGF group at the S and G2 phases.

As shown in Figure 3, the cell cycle progression data revealed that 15.40% of the cell population in the blank control group was in the S stage, while 23.85% and 17.41% of the HIOECs were in the S stage after treatment with 20 *μ*g/mL Nal-P-113 (*P*<0.05) and 10 ng/mL EGF (*P*>0.05). In addition, the percentages of HIOECs in the G0/G1 stage were 77.65%, 71.79%, and 75.27% in the blank control group, Nal-P-113 group, and rhEGF group, respectively. These results indicated that Nal-P-113 was able to promote the proliferation of HIOECs through facilitating the rapid entry of cells into the S phase and that the cells remained in this phase. Additionally, Nal-P-113 decreased the percentage of cells in the G2 stage compared to that in the blank control group (*P*<0.05). However, rhEGF did not accelerate the division of HIOECs.

### 3.4. RNA Expression of EGF, FGF-2, and TGF-*β*1

**Figure 4:**
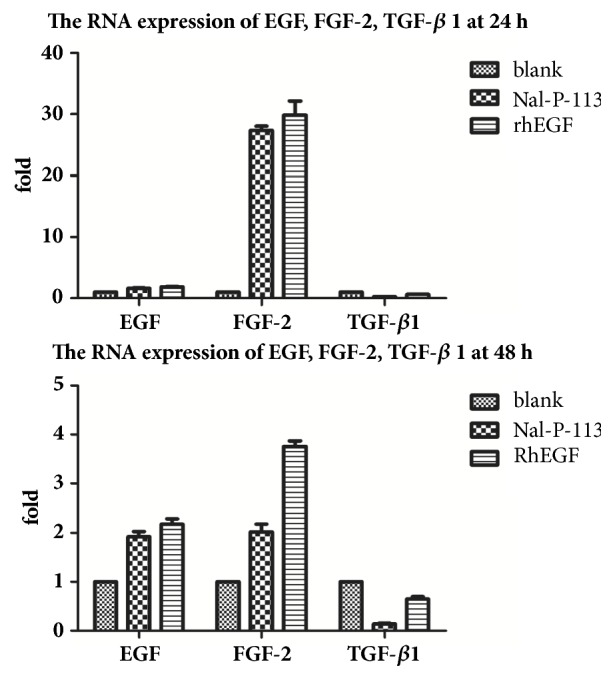
The RNA expression levels of EGF, FGF-2 and TGF-*β*1 were measured by quantitative RT-PCR when HIOECs were treated with blank control, 20 *μ*g/mL Nal-P-113 or 10 ng/mL rhEGF at 24 h and 48 h. 18s2 was included as an internal control, and the fold change was determined as the cytokine expression relative to that in the blank control group. The data are presented as the mean±SD of triplicate experiments.

The real-time PCR results showed that the expression levels of proliferation-related cytokines, including EGF, FGF-2, and TGF-*β*1, were similarly changed when cells were treated with 20 *μ*g/mL Nal-P-113 or 10 ng/mL rhEGF at 24 h and 48 h compared with the levels in the blank control group (*P* < 0.05). As shown in Figure 4, the RNA levels of EGF and FGF-2 were upregulated by 1.62- and 27.35-fold at 24 h in the presence of 20 *μ*g/mL Nal-P-113 in HIOECs. Additionally, the TGF-*β*1 RNA expression level was downregulated by 4.24-fold when HIOECs were treated with 20 *μ*g/mL Nal-P-113. Moreover, the above cytokines showed a similar trend of RNA expression levels in HIOECs in the presence of 20 *μ*g/mL Nal-P-113 at 48 h, though the differential multiplier of the FGF-2 RNA expression level was decreased.

### 3.5. Distribution of EGF, FGF-2, and TGF-*β*1 in Ulcer Loci of the Oral Mucosa

**Figure 5:**
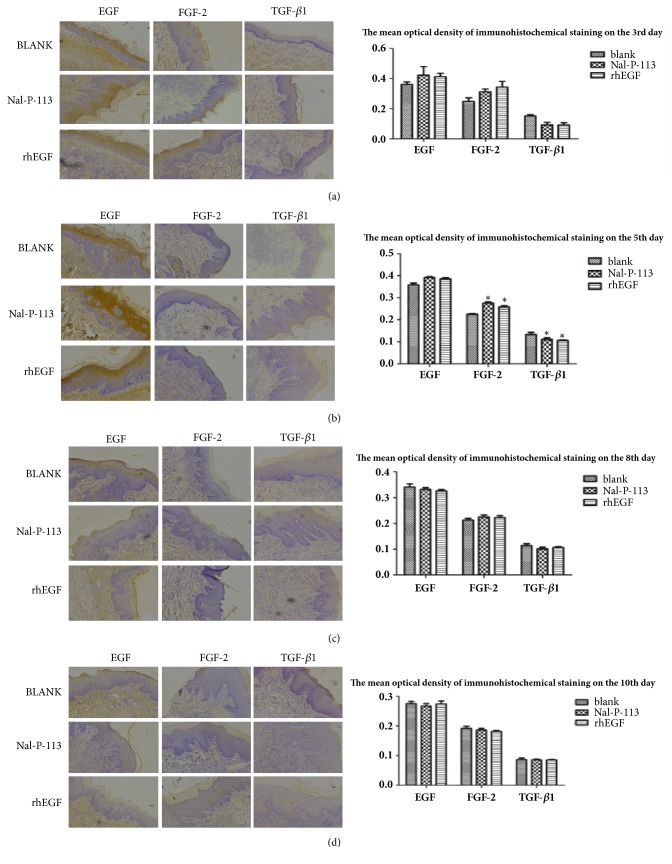
Expression of EGF, FGF-2, and TGF-*β*1 proteins in rat mucosal ulcer loci after treatment with blank control, 20 *μ*g/mL Nal-P-113 or 10 ng/mL rhEGF. (a) EGF, FGF-2, and TGF-*β*1 levels in rat mucosal ulcer loci on the 3^rd^ day after treatment with 20 *μ*g/mL Nal-P-113 or 10 ng/mL rhEGF compared with that in the blank control group. (b) EGF, FGF-2, and TGF-*β*1 levels in rat mucosal ulcer loci on the 5^th^ day after treatment with 20 *μ*g/mL Nal-P-113 or 10 ng/mL rhEGF compared with that in the blank control group. (c) EGF, FGF-2, and TGF-*β*1 levels in rat mucosal ulcer loci on the 8^th^ day after treatment with 20 *μ*g/mL Nal-P-113 or 10 ng/mL rhEGF compared with that in the blank control group. (d) EGF, FGF-2, and TGF-*β*1 levels in rat mucosal ulcer loci on the 10^th^ day after treatment with 20 *μ*g/mL Nal-P-113 or 10 ng/mL rhEGF compared with that in the blank control group. The immunohistochemical signal was measured by the Axioplan 2 imaging system. *∗*: *P* < 0.05.

To further evaluate the effect of Nal-P-113 on promoting cell proliferation in oral mucosal tissue* in vivo*, we established a mucosal ulcer model in rats to measure the expression changes in proliferation-related proteins, including EGF, FGF-2, and TGF-*β*1, with immunohistochemistry. The results showed that the expression levels of EGF and FGF-2 in the rat mucosal ulcer tissue were higher in the Nal-P-113 or rhEGF group than that in the blank control group, and TGF-*β*1 was the opposite (Figures 5(a) and 5(b), *P* < 0.05) on the 3^rd^ and 5^th^ days. However, the expression levels of EGF, FGF-2, and TGF-*β*1 between Nal-P-113 and rhEGF groups were roughly analogical. In addition, no significant difference in the expression of EGF, FGF-2, and TGF-*β*1 was observed on the 8^th^ and 10^th^ days when the ulcer loci of rats were treated with blank control, Nal-P-113 or rhEGF ((c) and (d) in Figure 5, *P* > 0.05).

## 4. Discussion

RAS is a highly prevalent self-healing oral mucosal disease that is characterized by a yellowish ulcer and may have a negative effect on the quality of life of the affected individual, impairing eating, swallowing, and speaking [29]. RAS ulcers usually develop on nonkeratinized oral mucosa, with the buccal and labial mucosa being the most common sites, and last for approximately 10-14 days without scar formation [30]. However, the underlying etiology and pathogenesis remain poorly understood. Therefore, the aims of treatment are to relieve pain, promote wound healing, and prevent secondary infection [31]. Many medications are clinically applied to treat oral mucosal ulcer, like analgesics, anaesthetics such as lidocaine, antibacterial agents, anti-inflammatory agents such as triclosan and diclofenac, steroids, sucralfate, a tetracycline suspension liquid and silver nitrate, natural herbal medicines, and so on [32]. Recently, CO_2_ laser, Nd:YAG laser, and diode laser have been also used to relieve some symptoms and promote the healing of wounds [33]. As for epidermal growth factor, some researches showed that reduced epidermal growth factor may increase the proliferation of human fibroblasts and keratinocytes, resulting in a susceptibility to the development of oral ulcers [34, 35]. Meantime, many clinicians remained concerned about the safety of rhEGF, citing fears that it could cause cancer happen. Up to now, no evidence has confirmed that rhEGF could induce the irregular proliferation of cells, but this risk must be considered before rhEGF is used for clinical applications [35]. Therefore, we conducted relevant research on the antimicrobial peptide Nal-P-113. Our previous study showed that Nal-P-113, a novel antimicrobial peptide, remained more than 85% intact in PBS, saliva, BHI medium, and bovine calf serum at 8 h and could effectively reduce oral bacteria partly when applied at a concentration of 20 *μ*g/mL* in vitro* and* in vivo* [9, 10]. Moreover, it might promote cells proliferate. In this study, 20 *μ*g/mL Nal-P-113 was chosen as an experimental agent, and 10 ng/mL rhEGF was used as a positive control agent to verify the effectiveness of Nal-P-113.

Our results from the CCK-8 and cell cycle assays revealed that 20 *μ*g/mL Nal-P-113 not only promoted the proliferation of HIOECs but also accelerated the entry of cells into the S phase of the cell cycle, which could increase the DNA replication of cells. The wound-healing assay verified that both Nal-P-113 and rhEGF facilitated cell migration compared with the blank control group at 48 h. The RT-PCR results suggested that Nal-P-113 and rhEGF were able to increase the RNA expression levels of EGF and FGF-2 and decrease that of TGF-*β*1 of HIOECs in 24 h and 48 h. EGF has been reported to be a major factor involved in the repair mechanism of ulcer healing and the management of the return to normal mucosal function [36–38]. Increased production of EGF in salivary glands is believed to be critical in the early phase of ulcer healing [37]. A low level of EGF results in a decreased healing capacity of the oral mucosa after injury [39, 40]. In particular, Fatimah SS et al. showed that EGF promoted the proliferation of human amnion epithelial cells through significantly increasing the proportion of the cells in the S and G2/M phases of the cell cycle [41]. Additionally, previous studies have indicated that both FGF-2 and TGF-*β*1 could regulate cell cycle progression by some cell signaling pathways [42–45]. Although it has been proved that Nal-P-113 and rhEGF had similar effects on HIOECs in many aspects, Nal-P-113 showed a great advantage in regulating the cell cycle of HIOECs, as indicated by the cell cycle assay. Moreover, combined with the RT-PCR results, these findings suggested that, on the one hand, Nal-P-113 could promote the differentiation and migration of HIOECs. On the other hand, Nal-P-113 increased the production of EGF and FGF-2 and decreased the production of TGF-*β*1 in HIOECs to hasten their entry into the S phase and accelerate the cell cycle, which are changes related to the ability of cytokines to facilitate cell growth and wound healing.

In the rat experiments, 20 *μ*g/mL Nal-P-113 and 10 ng/mL rhEGF promoted the expression of EGF and FGF-2 and reduced the TGF-*β*1 expression on the 5^th^ day. However, on the 8^th^ and 10^th^ day, the differences in EGF, FGF-2, and TGF-*β*1 expression in rat mucosal ulcers between the Nal-P-113 group or rhEGF group and the blank control group were not statistically significant (*P* > 0.05). During the first 5 days of ulcer formation, compared to physiological saline, Nal-P-113 promoted the secretion of a large amount of EGF and FGF-2 in the oral mucosa, but this effect was not observed after 5 days. This result suggested that Nal-P-113 could shorten the wound-healing time at the initial stage of ulcer healing. Therefore, there is reason to believe that the application of Nal-P-113 advanced the time at which HIOECs primarily produced EGF and FGF-2 and decreased TGF-*β*1, thus promoting oral ulcer healing.

Previous studies showed that some microbes or viruses are associated with RAS. When RAS was combined with the infection from other oral microbes, the patient had a longer healing time and aggravated pain. In addition to having an advantage over rhEGF in regulating the cell cycle, Nal-P-113 exerts a bactericidal effect that can also reduce bacterial infection and relieve the pain from the wound of the oral mucosa ulcer [9]. In addition, Nal-P-113 is a kind of gel formulation that is not easily diluted and easily adheres to the oral mucosal surface, thus readily insulating ulcer wounds from external irritation. With its combination of advantages, Nal-P-113 is indeed an effective method for treating RAS.

In summary, Nal-P-113 accelerates the cell cycle progression and promotes the synthesis of DNA by increasing the expression of the cell differentiation-related factors EGF and FGF-2 and reducing that of TGF-*β*1, thereby accelerating cell proliferation and wound healing. This agent has the potential to be used as a new therapeutic strategy for RAS in the clinic.

## 5. Conclusions

All the above experiments were conducted to assess the tissue reconstruction effect of Nal-P-113 on oral mucosa healing. The present study shows that Nal-P-113 can accelerate the proliferation of HIOECs and the repair of oral mucosal tissue damage in rats by regulating the cycle and migration of cells with changing the expression of cytokines related to cell propagation, which has wide applications in clinical treatment.

## Figures and Tables

**Table 1:** List of primer sequences used for RT-PCR.

Gene name	Primer (5′-3′)	Product size (bp)
FGF2	F: 5′ AAGAGCGACCCTCACATCA 3′	147
	R: 5′ AAAGAAACACTCATCCGTAACAC 3′	
TGF-*β*1	F: 5′ AAACCCACAACGAAATCTATGA 3′	280
	R: 5′ AACCACTGCCGCACAACT 3′	
EGF	F: 5′ GCTGCTCACTCTTATCAT 3′	283
	R: 5′ TCTTTCTAAATCCACCC 3′	
18s2	F: 5′ GAAACGGCTACCACATCC 3′	167
	R: 5′ ACCAGACTTGCCCTCCA 3′	

## Data Availability

The data used to support the findings of this study are available from the corresponding author upon request.
